# Ellagic Acid Prevents Particulate Matter-Induced Pulmonary Inflammation and Hyperactivity in Mice: A Pilot Study

**DOI:** 10.3390/ijerph20054523

**Published:** 2023-03-03

**Authors:** Sunyoung Jeong, Sungryong Bae, Eui-Cheol Shin, Jong-Hwa Lee, Jung-Heun Ha

**Affiliations:** 1 Bioanalytical and Pharmacokinetic Research Group, Korea Institute of Toxicology, Daejeon 34114, Republic of Korea; 2 Department of Human and Environmental Toxicology, University of Science and Technology, Daejeon 34113, Republic of Korea; 3 Department of Fire Protection and Disaster Management, Chosun University, Gwangju 61452, Republic of Korea; 4 Department of GreenBio Science/Food Science and Technology, Gyeongsang National University, Jinju 52725, Republic of Korea; 5 Department of Food Science and Nutrition, Dankook University, Cheonan 31116, Republic of Korea; 6 Research Center for Industrialization of Natural Neutralization, Dankook University, Yongin 16890, Republic of Korea

**Keywords:** particulate matter, ellagic acid, inflammation, hypoxia, hyperactivity

## Abstract

The inhalation of fine particulate matter (PM) is a significant health-related environmental issue. Previously, we demonstrated that repeated PM exposure causes hyperlocomotive activity in mice, as well as inflammatory and hypoxic responses in their lungs. In this study, we evaluated the potential efficacy of ellagic acid (EA), a natural polyphenolic compound, against PM-induced pulmonary and behavioral abnormalities in mice. Four treatment groups were assigned in this study (*n* = 8): control (CON), particulate-matter-instilled (PMI), low-dose EA with PMI (EL + PMI), and high-dose EA with PMI (EH + PMI). EA (20 and 100 mg/kg body weight for low dose and high dose, respectively) was orally administered for 14 days in C57BL/6 mice, and after the eighth day, PM (5 mg/kg) was intratracheally instilled for 7 consecutive days. PM exposure induced inflammatory cell infiltration in the lungs following EA pretreatment. Moreover, PM exposure induced inflammatory protein expression in the bronchoalveolar lavage fluid and the expression of inflammatory (tumor necrosis factor alpha (*Tnfα*), interleukin (*Il*)*-1b*, and *Il-6*) and hypoxic (vascular endothelial growth factor alpha (*Vegfα*), ankyrin repeat domain 37 (*Ankrd37*)) response genes. However, EA pretreatment markedly prevented the induction of expression of inflammatory and hypoxic response genes in the lungs. Furthermore, PM exposure significantly triggered hyperactivity by increasing the total moving distance with an increase in moving speed in the open field test. On the contrary, EA pretreatment significantly prevented PM-induced hyperactivity. In conclusion, dietary intervention with EA may be a potential strategy to prevent PM-induced pathology and activity.

## 1. Introduction

Air pollution continues to threaten public health in many cities and endangers the basic right to breathe. Diesel exhaust particles (DEPs) consist of a carbon core that adsorbs a mixture of sulfate, nitrate, metals, and organic chemicals, including polycyclic aromatic hydrocarbons (PAHs) and nitro-PAHs. DEPs are one of the major components of urban air pollution [1]. DEPs comprise mainly fine particulate matter (PM) with diameters less than 2.5 µm (PM 2.5), including nanoparticles, which can reach the lower lobe of the lung and even systemic circulation. Exposure to air pollutants, including DEPs, is inevitable, and there is cumulative evidence that indicates that continuous exposure to DEPs triggers detrimental effects on the pulmonary [2,3], renal [4,5], hepatic [6], cardiovascular [7,8], and nervous [9,10] systems. In particular, DEP inhalation significantly induces pulmonary inflammation, oxidative stress, and malfunction in mammals [11,12].

In addition, increased exposure to air pollutants triggers behavioral disorders in humans and experimental animals. The worsening degree of air pollution is closely intertwined with the early onset of attention deficit hyperactivity disorder in Taiwan [13,14]. In the US and Denmark, increased inhalation of air pollutants increases the incidence of psychiatric disorders, such as depression, bipolar disorder, and schizophrenia [15]. Although the exact developmental mechanisms of direct pathological causes of behavioral disorders are poorly understood, environmental challenges may be significant initiators of behavioral disorders [16,17]. In addition, in experimental mice, exposure of dams to air pollutants during pregnancy triggered hyperactivity in the pups [18,19]. Furthermore, we have previously demonstrated that PM instillation in relatively young adulthood (8~10 weeks) triggers hyperactivity in mice [20,21]. Interestingly, dietary intervention with phenolic components successfully prevented PM-induced hyperactivity in experimental mice [21].

If exposure to air pollution is inevitable, then dietary intervention with functional materials may be an excellent preventive means to attenuate and/or prevent air-pollutant-induced physiological disturbances [22,23,24,25]. Polyphenolic components are strong candidates for coping with exposure to air pollutants, given that polyphenols are abundant in plants and possess multiple biological functions, including anti-inflammatory [26,27,28], antiendoplasmic reticulum stress [29,30], and antioxidative effects [31,32,33]. Among polyphenols, ellagic acid (EA) may be a promising candidate to mitigate and/or prevent air-pollutant-induced pathophysiological responses in humans. EA is a conjugated form of two distinctive gallic acids, known as strong antioxidants, bridged by two lactone rings [34]. Plants (e.g., berries, grapes, and pomegranates) produce EA as a metabolite of tannin hydrolysis [34]. EA attenuates dyslipidemia [35], weight gain [36], insulin resistance [37], carcinogenesis [38,39], inflammatory responses [40,41], and oxidative stress [42,43]. Therefore, owing to its biological functionalities, EA may be a promising polyphenol candidate that can mitigate the effects of air pollutant inhalation.

EA is an excellent candidate for controlling pathophysiological phenomena during the inhalation of air pollutants. Inhalation of air pollutants directly induces pulmonary disturbances such as inflammation. Therefore, dietary supplements against exposure to air pollutants should be effective in mitigating pathological events in the lungs. According to previous reports, EA significantly ameliorated pulmonary damage triggered by various toxicants to the pulmonary system, such as hydrochloric acid [40], carbon tetrachloride [44], elastase [45], bleomycin with cyclophosphamide [46], and ovalbumin-induced asthma [47] in multiple animal models. The protective role of EA against pulmonary toxicants mainly relies on its anti-inflammatory and/or antioxidant effects [40,44,45,46,47]. Pretreatment with EA significantly attenuated LPS-induced acute pulmonary pathology and significantly reduced inflammatory cell infiltration and cytokine production (TNFα, IL-1β, and IL-6) in experimental mice [48].

Based on a literature review, EA may have protective functions against the effects of exposure of mammals to air pollutants (i.e., PM). However, animal models of PM exposure by instillation have only been established recently; therefore, robust experimental data are not yet available. Moreover, the preventive role of EA against pulmonary PM exposure has not yet been fully elucidated. In this study, to understand the protective effects of EA against acute pulmonary PM exposure, EA was orally administered at 20 and 100 mg/kg for 7 days before initiation of PM instillation. After 1 week of EA administration, PM (5 mg/kg) was instilled for 7 consecutive days while maintaining the aforementioned EA administration. To determine the beneficial effects of EA on PM exposure, pulmonary immune cell infiltration, PM loading, cytokine secretion, and mRNA expression were analyzed. Moreover, behavioral alterations caused by PM exposure and EA pretreatment were examined using an open field test (OFT).

## 2. Materials and Methods

### 2.1. Animal Experiments

All experimental animal procedures were previewed and approved by the Institutional Animal Care and Use Committee (protocol # 2002-0023) of the Korea Institute of Toxicology and accredited by the Association for Assessment and Accreditation of Laboratory Animal Care (AAALAC). Seven-week-old male C57BL/6NCrlOri mice (Orient Bio Inc., Seongnam, Republic of Korea) were acquired, acclimatized for 7 days, and maintained in a controlled room at a temperature of 22 °C and humidity of 50% with a 12 h light/dark cycle. The mice were allowed free access to a purified diet (PMI Nutrition International LLC, St. Louis, MO, USA) and filtered distilled water. After the acclimatization period, the experimental mice were weighed and randomly assigned to four groups (*n* = 8/group) as follows:

(1) Control (CON): 5% dimethyl sulfoxide (DMSO; Sigma-Aldrich, St. Louis, MO, USA) was administered orally for 14 days, and after the eighth day of DMSO administration, distilled water was instilled for another 7 days.
(2) PM-instilled (PMI): PM (5 mg/kg; standard reference material 2975; National Institute of Standards and Technology, Gaithersburg, MD, USA) was instilled for 7 days.
(3) Low-dose of EA with PMI (EL + PMI): EA (20 mg/kg; Sigma-Aldrich) was administered orally for 14 days, and after the eighth day of EA administration, PM (5 mg/kg) was instilled for another 7 days.
(4) High-dose of EA with PMI (EH + PMI): EA (100 mg/kg) was administered orally for 14 days, and after the eighth day of EA administration, PM (5 mg/kg) was instilled for another 7 days.

Fifteen days after the first EA administration, the mice were euthanized by isoflurane inhalation. After sacrifice, the final body, liver, and lung weights were measured. PM instillation was performed 1 h after EA treatment in the EL + PMI and EH + PMI groups.

### 2.2. Histological Analysis and Collection of Bronchoalveolar Lavage Fluid (BALF)

The left lung was fixed in 10% (*v*/*v*) neutral-buffered formalin (Sigma-Aldrich) and further processed for hematoxylin and eosin staining as previously described [20,21]. BALF was collected as previously described [20,21], and cells were counted using a cell counter (NC-250; ChemoMetec, Gydevang, Denmark). In addition, cell types in the BALF were distinguished after smearing with the cytospin slide (Thermo Fisher Scientific, Waltham, MA, USA) and staining with Diff-Quik solution (Dade Diagnostics, Aguada, Puerto Rico) as previously described [20,21].

### 2.3. Enzyme-Linked Immunosorbent Assay (ELISA)

Mouse TNFα (Invitrogen, Waltham, MA, USA), IL-6 (Invitrogen), and H_2_O_2_ (Biovision, Milpitas, CA, USA) levels in the BALF were analyzed using commercially available ELISA kits. Serum corticosterone levels were determined using an ELISA kit (Abcam, Cambridge, MA, USA). All ELISA procedures were performed in accordance with the manufacturer’s instructions.

### 2.4. Quantitative Real-Time PCR (qRT-PCR)

Total RNA extraction, cDNA synthesis, and relative qRT-PCR analyses were performed as previously described [21]. Primers used for qRT-PCR are indicated in Table 1 and previous literature [20,21].

### 2.5. Open Field Test

An open field test (OFT) was performed 1 h after the last PM instillation. Experimental mice were individually placed in the center of a plexiglass container (42 cm width × 42 cm depth × 42 cm height). The illumination of the plexiglass container was controlled by placing a 100 W lamp 2 m above the floor. The mice were acclimatized for 10 min in an OFT environment, and behavioral indices were recorded continuously for 10 min. Mice movements were recorded using an automated computer system (Ethovision, Noldus, The Netherlands). The distance, duration, and velocity of movements were calculated and expressed in inches, seconds, and inch/s, respectively.

### 2.6. Statistical Analysis

All data from the experiments are summarized and expressed as the mean ± standard deviation for each group. Equal variance of experimental data was assessed using the D’Agostino and Pearson omnibus test. If the datasets were normally distributed, one-way analysis of variance (ANOVA) with Tukey’s post hoc test was applied. Otherwise, Kruskal–Wallis with Dunn’s post hoc test was executed. Statistical significance was set at *p* < 0.05. Statistical analysis was performed using GraphPad PRISM 5 (GraphPad Software, San Diego, CA, USA).

## 3. Results

### 3.1. Alterations in Body and Relative Organ Weights

**Figure 1:**
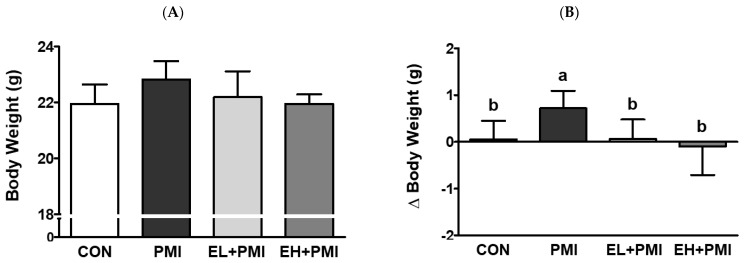

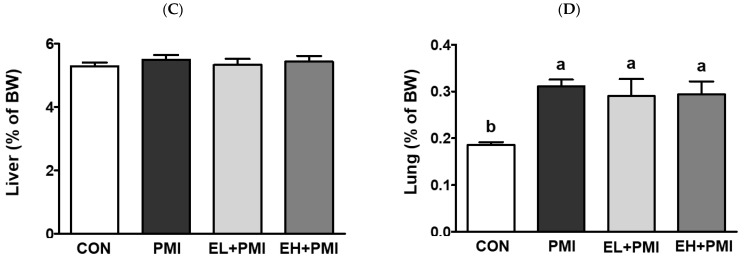
Body and relative weight changes in mice after ellagic acid (EA) pretreatment and particulate matter (PM) instillation. C57BL/6 mice were orally pretreated with ellagic acid (EL; 20 mg/kg or EH; 100 mg/kg) for 7 days, and then PM (5 mg/kg) was intratracheally administrated for 7 days with or without ellagic acid pretreatment: (**A**) body weight, (**B**) delta BW (final minus initial BW), (**C**) liver/body weight ratio, and (**D**) lung/body weight ratio. Values represent the mean ± standard deviation (SD) for each group (*n* = 8). Based on the D’Agostino and Pearson omnibus test, one-way ANOVA with Tukey’s post hoc test (for (**A**–**C**)) or Kruskal–Wallis test with Dunn’s post hoc test (for (**D**)) was applied. Lowercase letters indicate significant differences. *p* < 0.05.

Pulmonary PM exposure (PMI, EL + PMI, and EH + PMI) did not significantly alter the final body weight at sacrifice (Figure 1A). In contrast, delta body weight and relative lung weight were significantly increased by PM instillation (Figure 1B,D). Interestingly, EA treatment significantly attenuated the delta body weight against PM exposure regardless of the EA concentration (Figure 1B). However, PM-induced lung weight did not change with EA treatment (Figure 1D). There were no significant differences in the relative liver weight among the experimental groups (Figure 1C). EA treatment did not affect the final body weight or the relative lung and liver weights. The overall changes in patterns of delta body weight and relative lung weight exhibited similar trends to our previous studies on quercetin treatment [21]. In our previous findings, PM instillation increased delta body weight [20,21]; however, quercetin treatment inhibited the PM-induced increase in delta body weight [21], similar to the effect exerted by EA treatment in the current study.

### 3.2. Pulmonary PM Loading and Inflammatory Cytokine Secretion

**Figure 2:**
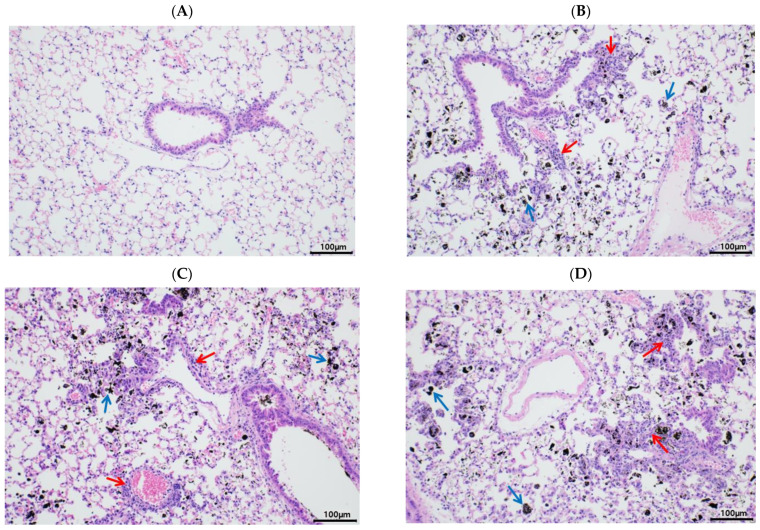
Histological changes in lung tissue after ellagic acid (EA) pretreatment and particulate matter (PM) instillation. C57BL/6 mice were orally pretreated with ellagic acid (EL; 20 mg/kg or EH; 100 mg/kg) for 7 days, and then PM (5 mg/kg) was intratracheally administrated for 7 days with or without ellagic acid pretreatment. Representative images of hematoxylin and eosin (H & E) stained sections obtained from the (**A**) CON, (**B**) PMI, (**C**) EL + PMI, and (**D**) EH + PMI groups (*n* = 8). Blue arrows indicate black-pigment-laden macrophages in the alveoli and alveolar lumen interstitium. Red arrows point to the infiltrated inflammatory cells in the peribronchiolar, perivascular, and interstitial regions. Scale bars = 100 μm.

**Figure 3:**
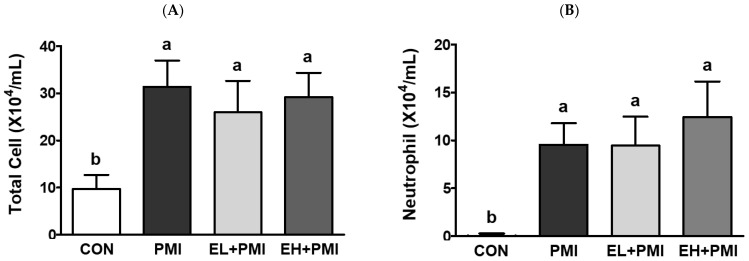

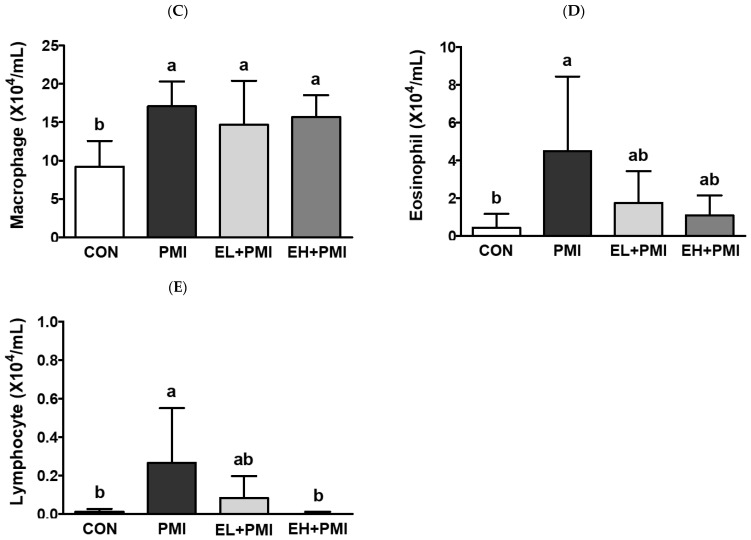
Inflammatory cell profiles in the bronchoalveolar lavage fluid (BALF) after ellagic acid (EA) pretreatment and particulate matter (PM) instillation. C57BL/6 mice were orally pretreated with ellagic acid (EL; 20 mg/kg or EH; 100 mg/kg) for 7 days, and then PM (5 mg/kg) was intratracheally administrated for 7 days with or without ellagic acid pretreatment. All the cells in the BALF were stained with Diff-Quik solution and counted: (**A**) total cells, (**B**) neutrophils, (**C**) macrophages, (**D**) eosinophils, and (**E**) lymphocytes. Values represent the mean ± standard deviation (SD) for each group (*n* = 8). Based on the D’Agostino and Pearson omnibus test, one-way ANOVA with Tukey’s post hoc test (for (**A**–**C**,**E**)) or Kruskal–Wallis test with Dunn’s post hoc test (for (**D**)) was applied. Lowercase letters indicate significant differences. *p* < 0.05.

According to our previous findings, PM instillation directly induces pulmonary inflammatory responses in rodents [20]. Similar to our previous findings, in this study, PM exposure resulted in black particles or black pigment-laden alveolar macrophages in the alveolar areas (Figure 2B, blue arrows). In addition, infiltrated inflammatory cells were noted in the peribronchiolar, perivascular, and interstitial regions (Figure 2B, red arrows), similar to our previous reports [20]. Pulmonary PM loading induces infiltration of immune cells and cytokine secretion in the BALF [20], and we postulated that EA treatment would attenuate the recruitment of immune cells and cytokine secretion in the BALF. As expected, PM exposure markedly increased the total number of immune cells and inflammatory cytokines in the BALF. After PM exposure, total immune cells in the BALF were increased ~3-fold compared with CON with induction of the absolute number of neutrophils and macrophages (Figure 3A–C). In addition, a significant increase in the number of eosinophils and lymphocytes was induced in the PMI group compared with the CON group (Figure 3D,E). However, the total number of immune cells was not ameliorated in the EL + PMI and EH + PMI groups compared with the PMI group (Figure 3A–C), similar to the results in our previous study on quercetin treatment [21]. In contrast, the number of eosinophils and lymphocytes gradually decreased in the EA-treated groups compared with that in the PMI group in an EA dose-dependent manner (Figure 3D,E).

**Figure 4:**
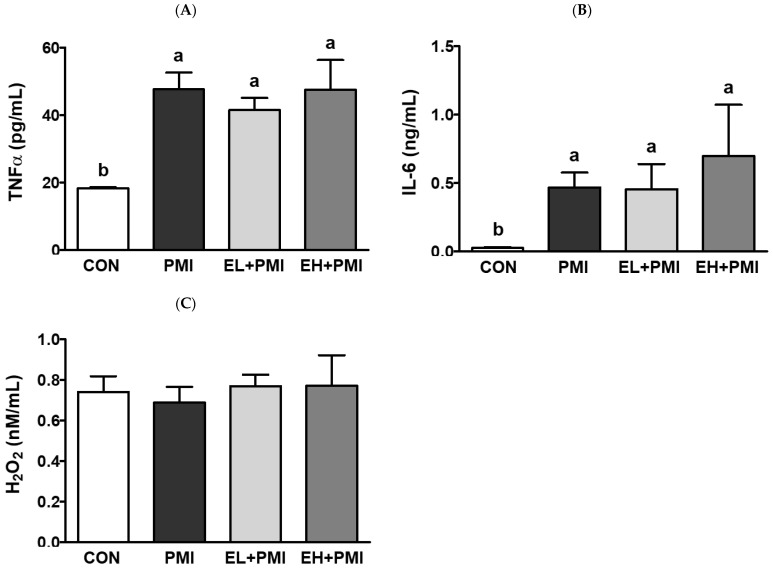
Inflammatory cytokine and H_2_O_2_ levels in the BALF after ellagic acid (EA) pretreatment and particulate matter (PM) instillation. C57BL/6 mice were orally pretreated with ellagic acid (EL; 20 mg/kg or EH; 100 mg/kg) for 7 days, and then PM (5 mg/kg) was intratracheally administrated for 7 days with or without ellagic acid pretreatment: (**A**) TNFα, (**B**) IL-6, and (**C**) H_2_O_2_ levels in the BALF. Values represent the mean ± standard deviation (SD) for each group (*n* = 8). Data passed the D’Agostino and Pearson omnibus normality test; one-way ANOVA with Tukey’s post hoc test was applied. Lowercase letters indicate significant differences. *p* < 0.05.

PM exposure also upregulated pulmonary cytokine secretion in the BALF. After PM instillation, pulmonary TNFα and IL-6 protein secretion was remarkably elevated, consistent with our previous findings [20,21]. PM exposure elevated pulmonary TNFα and IL-6 secretion in the BALF by approximately 2.6- and 19-fold, respectively, compared with the CON group (Figure 4A,B). However, EA treatment did not prevent the induction of pulmonary inflammatory cytokine secretion in the BALF (Figure 4A,B). In our previous study, quercetin treatment also failed to prevent PM-induced recruitment of immune cells and cytokine secretion in the BALF [21]. Therefore, PM instillation may directly and strongly induce pulmonary inflammation, and EA and quercetin [21] may not fully prevent inflammatory events such as physical PM loading and inflammatory cytokine secretion in the BALF. Moreover, we measured hydrogen peroxide levels in the BALF to determine whether PM exposure may induce pulmonary oxidative stress. PM exposure for 1 week did not increase hydrogen peroxide secretion in the BALF (Figure 4C), consistent with our earlier findings [20,21].

### 3.3. EA Treatment Prevented PM-Induced Expression of Inflammatory and Hypoxic Response Genes

**Figure 5:**
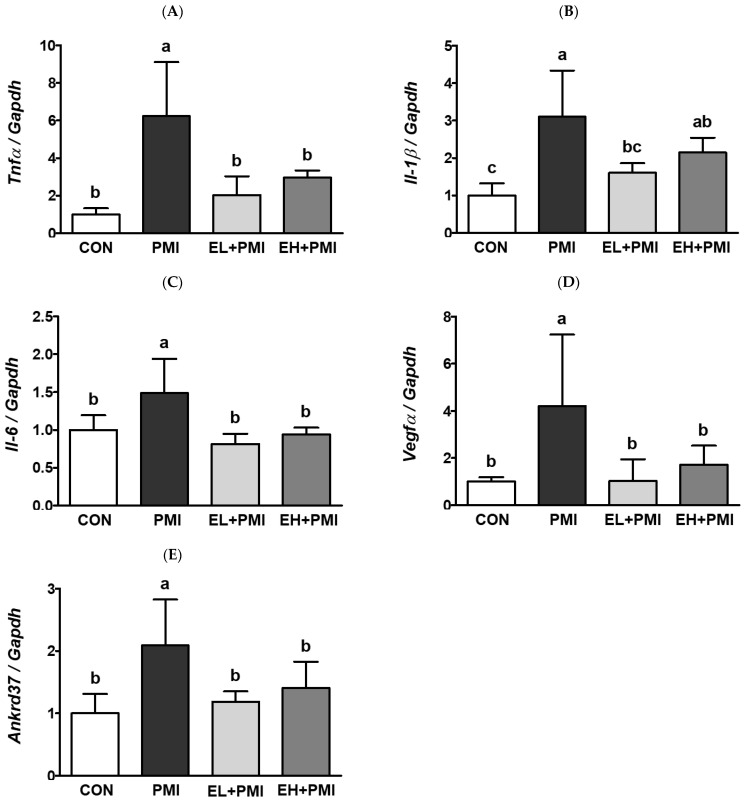
Pulmonary mRNA expressions after ellagic acid (EA) pretreatment and particulate matter (PM) instillation. C57BL/6 mice were orally pretreated with ellagic acid (EL; 20 mg/kg or EH; 100 mg/kg) for 7 days, and then PM (5 mg/kg) was intratracheally administrated for 7 days with or without ellagic acid pretreatment: (**A**) *Tnfα*, (**B**) *Il-1β*, (**C**) *Il-6*, (**D**) *Vegfα*, and (**E**) *Ankrd37* expression was analyzed using qRT-PCR normalized to *Gapdh* mRNA expression. Values represent the mean ± standard deviation (SD) for each group (*n* = 8). Data passed the D’Agostino and Pearson omnibus normality test; one-way ANOVA with Tukey’s post hoc test was applied. Lowercase letters indicate significant differences. *p* < 0.05.

EA treatment did not reduce the recruitment of immune cells or cytokine secretion in the BALF. However, in our previous study, we observed that quercetin exerted anti-inflammatory effects by decreasing pulmonary cytokine mRNA expression [21]. Similarly, pulmonary cytokine mRNA expression was increased in the PMI group compared with that in the CON group. The mRNA expression of pulmonary cytokines such as *Tnfα*, *Il-1b,* and *Il-6* increased 6.2-, 3.1-, and 1.4-fold, respectively, in the PMI group compared with the CON group (Figure 5A–C). However, EA treatment decreased PM-induced pulmonary *Tnfα* mRNA expression by 1.9-fold and 2.9-fold in the EL + PMI and EH + PMI groups, respectively, compared with the PMI group (Figure 5A). In addition, EA treatment remarkably reduced PM-induced pulmonary *Il-1b* mRNA expression by 1.6-fold and 2.1-fold in the EL + PMI and EH + PMI groups, respectively, compared with the PMI group (Figure 5B). Furthermore, EA treatment also reduced PM-induced pulmonary *Il-6* mRNA expression by 0.8-fold and 0.9-fold in the EL + PMI and EH + PMI groups, respectively, compared with the PMI group (Figure 5C).

Inflammatory and hypoxic responses are often coincidental physiological events that occur in a site- and cell-type-specific manner [49]. To understand whether (1) PM exposure induced pulmonary hypoxic responses and (2) EA treatment prevented PM-induced hypoxic responses, mRNA expression of hypoxic response genes (e.g., *Vegfα* and *Ankrd37*) was assessed in the lung tissue by qRT-PCR. As expected, pulmonary *Vegfα* mRNA expression in the PMI group was elevated 4.2-fold compared with that in the CON group; however, pulmonary *Vegfα* mRNA expression was markedly attenuated by 1- and 1.7-fold in the EL + PMI and EH + PMI groups, respectively, compared with that in the PMI group (Figure 5D). In addition, pulmonary *Ankrd37* mRNA expression in the PMI group increased by 2.1-fold compared with the CON group; however, pulmonary *Ankrd37* mRNA expression was significantly attenuated by 1.2- and 1.4-fold in the EL + PMI and EH + PMI groups, respectively, compared with the PMI group (Figure 5E). Similar trends were also observed in a previous experiment in which PM exposure elevated the mRNA expression of genes for an inflammatory and hypoxic response, which was significantly reduced by quercetin treatment [21].

### 3.4. EA Treatment Prevented PM-Induced Hyperactivity

**Figure 6:**
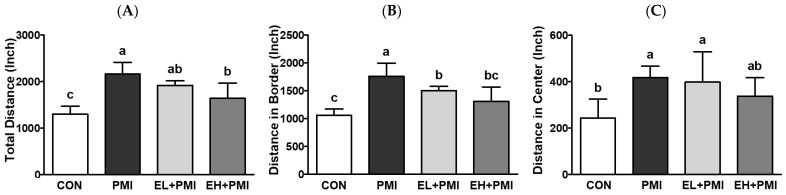

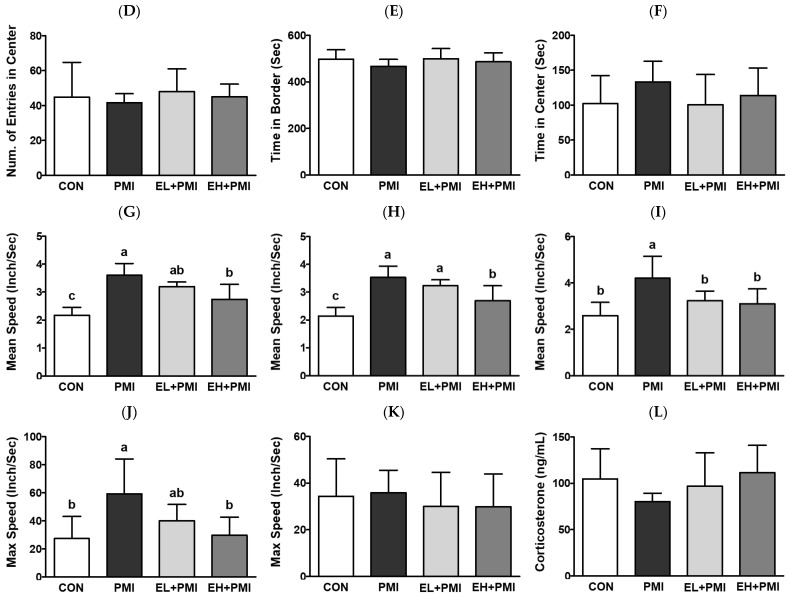
Open field test results and serum corticosterone levels after ellagic acid (EA) pretreatment and particulate matter (PM) instillation. C57BL/6 mice were orally pretreated with ellagic acid (EL; 20 mg/kg or EH; 100 mg/kg) for 7 days, and then PM (5 mg/kg) was intratracheally administrated for 7 days with or without ellagic acid pretreatment: (**A**) total distance moved, (**B**) distance moved in border, (**C**) distance moved in center, (**D**) number of entries into center, (**E**) time spent in border, (**F**) time spent in center, (**G**) the total mean speed, (**H**) mean speed in border, (**I**) mean speed in center, (**J**) max speed in border, (**K**) max speed in center of mice within 10 min of the open field test, and (**L**) serum corticosterone measured 1 day after the OFT. Values represent the mean ± standard deviation (SD) for each group (*n* = 8). Data passed the D’Agostino and Pearson omnibus test; one-way ANOVA with Tukey’s post hoc test was applied. Lowercase letters indicate significant differences. *p* < 0.05.

To evaluate the behavioral effects of EA on PM-exposed mice, an OFT was implemented. In this study, mice in the PMI group exhibited hyperactivity compared with the CON group. The total moving distance, including both the outer and central parts of the plexiglass container, were increased in the PMI group compared with the CON group (Figure 6A–C). Interestingly, PM-treated mice spent a significantly increased amount of time in the central area, whereas quercetin treatment decreased [21]; however, in this experiment, all treatments did not significantly alter the time spent in the central part of the plexiglass container (Figure 6F). Although there were no statistical differences, PM exposure increased the time spent in the central part of the plexiglass container by approximately 30.4% (*p* = 0.19) compared with the CON group, while EL + PMI and EH + PMI decreased the central staying time by approximately 75.5% and 85.6%, respectively, compared with the PMI group. In conjunction with, in all groups, no significant changes were noted in the staying time on the border of the plexiglass container (Figure 6E). The total movement speed of the PMI group was significantly elevated with hyperactivity (mean and maximum speed) at the border of the plexiglass container (Figure 6G,H,J). For the PMI group, the maximum speed at the central area did not significantly increase (Figure 6K); however, the mean speed at the central area significantly increased (Figure 6I). In contrast, in the EL + PMI and EH + PMI groups, the hyperactivity observed in the PMI group was reduced. Subsequently, we proposed that amelioration of PM-induced hyperactivity with EA treatment may involve serum corticosterone, a gold standard to assess stress levels. To answer our extended research question, serum corticosterone levels were analyzed using an ELISA kit. However, there were no distinguishable differences in serum corticosterone levels among the experimental groups (Figure 6L), as in our previous study [21].

## 4. Discussion

In this study, we investigated the potential protective effects of EA against PM-induced pulmonary pathology and locomotor hyperactivity in experimental rodents. PM exposure is an inevitable and chronic event; therefore, dietary intervention with supplementation of functional phenolic compounds may be an ideal means to prevent and/or attenuate PM-induced pulmonary pathology and behavioral alterations. To understand whether EA pretreatment effectively attenuated PM-induced pulmonary pathology and hyperactivity, we used our previous pilot experimental conditions [20,21]. Briefly, mice were supplemented with vehicle control or EA (20 or 100 mg/kg) for 7 days, and then PM was instilled with continuous dietary interventions for the following 7 days. Pulmonary PM loading was a physical and inevitable event because EA pretreatment failed to prevent pulmonary PM accumulation and recruitment of immune cells in the BALF. EA pretreatment partially prevented PM-induced pulmonary cytokine and hypoxic mRNA expression and hyperactivity.

PM instillation significantly elevated PM loading in the lung and pulmonary inflammatory responses, similar to our previous findings [20,21]. Based on the histological evaluation, black materials from the PM were markedly accumulated in the alveolar lumen and interstitial tissue in all PMI groups, regardless of the EA pretreatment. In the BALF, PM instillation significantly induced infiltration of immune cells such as neutrophils and macrophages, as noted in previous publications [20,21]. Moreover, cytokine secretions in the BALF, such as those of IL-6 and TNFα, were remarkably elevated in all PMI groups. EA treatment did not significantly prevent IL-6 and TNFα induction in the BALF. Jeong et al. also reported that dietary intervention with quercetin did not prevent PM loading in the lung and cytokine secretion in the BALF [21]. Probably, the PM loading concentration in our experimental protocol was in excess, as evidenced by pulmonary PM loading; therefore, dietary intervention may not be sufficient to prevent pulmonary cytokine secretions in BALF.

However, EA pretreatment significantly attenuated PM-induced pulmonary cytokine and hypoxic mRNA expression in our experiments. As expected, PM instillation significantly induced the mRNA expression of pulmonary cytokines (*Il-1b*, *Tnfα*, and *Il-6*), as increased cytokine secretion was observed in the BALF. Moreover, the expression of hypoxic response genes (e.g., *Ankrd37* and *Vegfα*) was markedly elevated in the PMI group. The induction of inflammatory and hypoxic responses verified our previous results [21]. However, EA treatment significantly reduced PM-induced inflammatory and hypoxic changes in mRNA expression. Key regulatory proteins for inflammatory and hypoxic responses are NF-κB and HIF1α, respectively, which are closely intertwined at the molecular level [50]. The NFκB and HIF1α pathways share a common molecular denominator, the IKK complex; therefore, the induction of NFκB by phosphorylation may trigger hypoxic signal induction of HIF1α, and vice versa. Our previous [21] and current findings suggest that dietary intervention with phenolic compounds (e.g., quercetin and EA) may attenuate PM-induced pulmonary inflammatory and hypoxic mRNA expression. In future studies, the expression of NFκB and HIF1α pathways should be scrutinized to understand whether dietary intervention can prevent PM-induced pulmonary inflammation and/or hypoxic events.

EA pretreatment significantly attenuated PM-induced locomotor hyperactivity in experimental mice. The PMI group had increased total, border, and center moving distances and mean speeds and increased maximum speed at the border compared with the CON group. Interestingly, EA pretreatment decreased the distinctive PM-induced hyperactivity by attenuating moving distances in total (EH + PMI), border (all EA treatments), and center (EH + PMI), mean speeds in total (EH + PMI), border (EH + PMI), and center (all EA treatments), and maximum speed in the border (EH + PMI). Previous findings using cohort studies have also demonstrated that PM exposure in early developmental periods triggers attention deficit hyperactivity disorder-like hyperactivity [14,51]. In addition, high-DEP exposure prenatally and 1 week after birth led to increased hyperactivity in experimental mice [18]. Moreover, maternal PM exposure significantly triggered hyperactivity in pups in a mouse model [19]. In this study, we demonstrated that PM instillation in relatively young adulthood (8~10 weeks) also increased locomotor activity in mice, consistent with our previous findings [20,21]. Interestingly, dietary intervention with phenolic components, such as EA and quercetin [21], successfully prevented PM-induced hyperactivity in experimental mice. An increased chance of inhalation of air pollutants is closely intertwined with an elevation in abnormal behaviors, such as depression, bipolar disorder, and schizophrenia [15]. Therefore, finding and applying functional dietary resources (e.g., EA and quercetin) as preventive measures against air pollutants may be a possible and sustainable strategy to maintain normal health.

Our current findings have significant advantages and disadvantages when extrapolating to the clinical field. Our experimental conditions included limited dietary intervention, PM exposure time, and PM concentration. Exposure of humans to PM may be long-term; however, our experimental protocol was executed in a relatively short-term period (14 days of dietary intervention and 7 days of PM exposure) with relatively higher concentrations of PM. Dietary intervention with phenolic compounds (EA and quercetin [21]) did not significantly prevent inflammatory cytokine secretion in the BALF. It seems that our experimental conditions may not fully account for potential pathological events and dietary interventions in humans. In addition, we detected hydrogen peroxide to gauge the pulmonary oxidative stress level in the BALF because prolonged inflammation may induce oxidative stress. Under hypoxic conditions, oxidative stress is generally elevated by ROS induction of reactive oxygen species [52]. Therefore, we postulated that hydrogen peroxide would be increased by PM exposure because of the induction of hypoxic *Ankrd37* and *Vegfa* mRNA expression in the lungs. However, hypoxic mRNA expression in the lung and hydrogen peroxide secretion in the BALF did not match because hydrogen peroxide concentrations in the BALF were similar among all experimental groups. In future studies, we need to optimize the experimental conditions to make robust conclusions regarding whether PM exposure triggers pulmonary hypoxic responses. In addition, hyperactivity was noted in the PMI group, but EA pretreatment significantly normalized hyperactivity in mice. Our previous study used an identical experimental setting; quercetin also prevented PM-induced hyperactivity [21]. Therefore, we hypothesized that the stress hormone corticosterone would be altered by PM exposure; however, serum corticosterone levels were unchanged among all treatments, regardless of dietary intervention or PM treatment. Therefore, in the future, we may try to find any behavior-related hormones that are controlled by PM exposure and dietary intervention.

Although there are restrictions, there are numerous advantages to our experimental setting. In our current and previous experiments [21], in a relatively short period of time, we remarkably observed the preventive potency of EA and quercetin [21] against pulmonary inflammatory and hypoxic mRNA expression induced by PM exposure. Therefore, in the future, the application of optimized and lower PM concentrations to reflect current air pollution with longer experimental periods may result in the positive suppression of PM-induced infiltration of inflammatory cells and cytokine secretion in the BALF. Another promising finding was the behavioral alterations observed in our mouse model. Similar to other PM exposure models in the early life phases [18,19,53], we also found that PM exposure in early adulthood induced hyperactivity in mice. A relatively short period of dietary intervention with EA and quercetin [21] effectively normalized hyperlocomotive activity. Therefore, dietary intervention may be an acceptable approach for maintaining normal behavior amidst PM exposure.

EA is a widely accepted dietary polyphenol with multiple beneficial effects, especially in reducing biological inflammatory reactions [41,54,55,56]. In our experiments, EA pretreatment prevented PM-induced pulmonary cytokine mRNA expression over a relatively short period (14 days). Other studies have demonstrated that EA has significant efficacy in attenuating pulmonary inflammation, oxidative stress, and fibrosis (Table 2). In an acute lung injury (ALI) mouse model triggered by hydrochloric acid, oral EA treatment reduced neutrophil recruitment in the BALF and the lungs [40]. In this model, EA decreased the proinflammatory cytokine IL-6 and increased the anti-inflammatory cytokine IL-10 in the BALF [40]. In addition, EA treatment exerted an anti-inflammatory effect in an LPS-induced ALI model [48]. EA treatment also attenuated elastase-induced immune cells and cytokine secretion in the BALF in an emphysema model [45]. In a murine asthma model, EA treatment also prevented pulmonary inflammation by suppressing pulmonary NFκB activation [47]. Furthermore, EA has anti-inflammatory, antioxidative [44,46], and antifibrosis effects [46] in experimental rodents.

In this study, EA pretreatment significantly prevented PM-induced pulmonary inflammatory and hypoxic mRNA expression, along with the normalization of hyperlocomotive activity. However, inflammatory cytokine and hydrogen peroxide secretion in the BALF did not alter with either PM exposure or EA pretreatment. Our study is a novel endeavor in at least two aspects: (1) investigating the pulmonary pathophysiology of PM instillation and (2) investigating whether dietary intervention with EA could thwart PM-induced pathology. To date, dietary preventive means in PM-exposed animal experiments have just begun [21]; therefore, there is limited information on which experimental settings are suitable for potential clinical application. Our experimental period may have been relatively short, considering PM exposure in humans has a longer incidence. We also used a relatively higher PM concentration compared with those that humans are practically exposed to. Therefore, in future studies, we may optimize our experimental protocols by increasing the PM exposure duration and using lower PM concentrations. Although our experimental setting has some limitations, prevention of pulmonary inflammatory and hypoxic mRNA expression by EA pretreatment may also prevent PM-induced protein expression and function. Another obvious finding was that dietary intervention with EA pretreatment normalized PM-induced hyperactivity.

## 5. Conclusions

This study investigated the effectiveness of EA, a natural polyphenolic compound, in preventing the adverse effects of PM exposure in C57BL/6 mice. Four groups of mice were assigned (CON, PMI, EL + PMI, and EH + PMI); EA was orally administered for 14 days in C57BL/6 mice, and after the eighth day, PM (5 mg/kg) was intratracheally instilled for 7 consecutive days. The experimental results demonstrated that EA pretreatment with EA prevented PM-inducible pulmonary inflammatory and hypoxic mRNA induction, as well as hyperactivity in the experimental mice. This study suggests that EA may be a promising approach for mitigating the pathophysiological impacts of PM exposure.

## Figures and Tables

**Table 1:** Primer sequences.

Transcript	Forward Primer (5′ to 3′)	Reverse Primer (5′ to 3′)
*Tnfα*	GGCTGCCCCGACTACGT	ACTTTCTCCTGGTATGAGATAGCAAAT
*Il-1β*	GTCACAAGAAACCATGGCACAT	GCCCATCAGAGGCAAGGA
*Il-6*	CTGCAAGAGACTTCCATCCAGTT	AGGGAAGGCCGTGGTTGT
*Vegfα*	GAGCAGAAGTCCCATGAAGTG	TGTCCACCAGGGTCTCAATC
*Ankrd37*	CGGCCTTGCGTGCTTT	TGGTTGAGGTCAGCACCTGTT
*Gapdh*	CATGGCCTTCCGTGTTCCTA	GCGGCACGTCAGATCCA

**Table 2:** Beneficial effects of ellagic acid (EA) in pulmonary pathology in experimental rodents.

Strain	Model/Inducer	EA Treatment	Biological Marker	Ref.
BALB/c mice	Acute lung injury/hydrochloric acid	10 mg/kg, p.o.	BALF and lung neutrophil ↓	[40]
			BALF IL-6 ↓	
			BALF IL-10 ↑	
BALB/c mice	Acute lung injury/LPS	5 mg/kg, i.p.	BALF TNFα, IL-1β, IL-6 ↓	[48]
			BALF IL-10 ↑	
			BALF total protein ↓	
			Lung myeloperoxidase ↓	
Sprague Dawley rats	Emphysema/elastase	30 mg/kg, p.o.	Lung edema ↓	[45]
			Lung immune cell infiltration ↓	
			BALF immune cells ↓	
			BALF TNFα and IL-6 ↓	
BALB/c mice	Asthma/ovalbumin	10 mg/kg, p.o.	BALF eosinophils ↓	[47]
			Mucus production ↓	
			BALF IL-4, IL-5, IL-13 ↓	
			Lung NFκB activation ↓	
Wistar rats	Oxidative stress/carbon tetrachloride	10 mg/kg, i.p.	Lung malondialdehyde ↓	[44]
			Lung catalase activity, glutathione, Nrf-2 ↑	
			Lung NFκB, COX-2, TNFα ↓	
Wistar rats	Fibrosis-like/bleomycin and cyclophosphamide	15 mg/kg, p.o.	Lung hydroxyproline ↓	[46]
			Lung lipid peroxidation ↓	
			Lung glutathione, antioxidant enzymes ↑	
			Lung myeloperoxidase ↓	
			BALF immune cell, total protein ↓	

Abbreviations: BALF, bronchoalveolar lavage fluid; COX-2, cyclooxygenase 2; i.p., intraperitoneal; IL-1β, interleukin-1 beta; IL-4, interleukin-4; IL-5, interleukin-5; IL-6, interleukin-6; IL-10, interleukin-10; IL-13, interleukin-13; LPS, lipopolysaccharide; NFκB, nuclear factor kappa B, p65; Nrf-2, nuclear factor erythroid 2-related factor 2; p.o., per oral; TNFα, tumor necrosis factor alpha; ↓ decreased; ↑ increased.

## Data Availability

The data presented in this study are available from the corresponding authors upon request.
